# Power-response fingerprinting reveals probe-dependent scaling and a productivity-efficiency trade-off in a high-frequency sonoreactor

**DOI:** 10.1016/j.ultsonch.2026.108086

**Published:** 2026-09-29

**Authors:** Oualid Hamdaoui, Hamza Ferkous, Muthupandian Ashokkumar

**Affiliations:** a Chemical Engineering Department, College of Engineering, King Saud University, 12372 Riyadh, Saudi Arabia; b Department of Process Engineering, Faculty of Technology, Badji Mokhtar - Annaba University, 23000 Annaba, Algeria; c School of Chemistry, The University of Melbourne, Parkville 3010, Victoria, Australia

**Keywords:** High-frequency ultrasound, Multi-dosimetry, Power-response fingerprinting, Energy-specific response, Hydrogen peroxide, Dissolved gas

## Abstract

Chemical dosimetries are widely used to characterize acoustic cavitation, yet their signals are often treated as interchangeable measures of a single radical yield. The unresolved question is whether differences among these signals remain as fixed assay-specific offsets or change systematically when acoustic power is varied. Here, power-response fingerprinting (PRF) is introduced as a probe-aware framework for comparing how chemically distinct reporters respond to the same calorimetrically measured power perturbation. At 585 kHz, the dissipated acoustic power was 9.65, 38.0, and 79.0 W, and the corresponding responses were quantified using potassium iodide (KI/I_3_^−^), hydrogen peroxide (H_2_O_2_), Fricke (Fe3⁺), and 4-nitrophenol hydroxylation to 4-nitrocatechol (4-NC). An 8.19-fold increase in calorimetric power increased the formation rates by only 5.13-fold for KI, 5.67-fold for H_2_O_2_, 3.06-fold for Fricke, and 3.12-fold for 4-NC, giving descriptive global power exponents of 0.74, 0.80, 0.49, and 0.51, respectively. Energy normalization revealed a complementary productivity-efficiency trade-off: at 79 W, the responses per joule retained only 0.63, 0.69, 0.37, and 0.38 of their 9.65 W values, and the largest decrease occurred between 9.65 and 38 W. At the common high-power generator setting, Ar increased H_2_O_2_ accumulation to 1.88 times the unsparged-water rate, whereas N_2_ reduced it to 0.16 times that rate. The matched response vector therefore shows that acoustic power changes the relative expression of measurable sonochemical channels rather than uniformly amplifying a universal cavitation output. PRF separates chemical productivity from energy-specific performance without treating chemically different assays as stoichiometrically equivalent.

## 1 Introduction

Acoustic cavitation converts an externally imposed pressure field into transient, spatially heterogeneous physicochemical microenvironments. Bubble growth and collapse can generate extreme local temperatures and pressures, dissociate water vapor, and initiate radical and molecular reaction networks involving HO^•^, H^•^, oxygen-centered radicals, and H_2_O_2_ [1], [2], [3]. In a practical multibubble reactor, however, the measured chemical output is an ensemble property rather than a direct readout of an individual collapse. It reflects the coupled effects of bubble nucleation, population density, interbubble interaction, acoustic attenuation, mass transfer, streaming, and non-cavitational dissipation. Consequently, two operating states with similar total dissipated power need not produce the same chemical field, and no single global observable can be assumed to represent cavitation chemistry in all reaction matrices.

Calorimetry and chemical dosimetry interrogate different levels of this hierarchy. Calorimetry provides an experimentally accessible measure of the power dissipated in the liquid and replaces equipment-specific amplitude settings with an energetic basis [4]. Chemical dosimetries report the consequences of cavitation through different reaction networks. KI integrates iodide-oxidizing pathways; the Fricke system reports chain oxidation of Fe^2^⁺ in a strongly acidic matrix; H_2_O_2_ represents net molecular accumulation after concurrent formation and consumption; and conversion of 4-nitrophenol (4-NP) to 4-nitrocatechol (4-NC) samples one identifiable aromatic hydroxylation branch [[5], [6], [7], [8]]. Classical and comparative dosimetry studies have likewise shown that iodide, Fricke, terephthalate, salicylate, H_2_O_2_, and related probes differ in stoichiometry, selectivity, and the reaction zones that they sample [[9], [10], [11], [12], [13]]. Their signals are therefore complementary rather than stoichiometrically interchangeable estimates of a universal HO^•^ concentration.

This distinction becomes critical when acoustic power is varied. Increasing power can activate additional nuclei and increase bubble excursion, but the chemically productive fraction of the added energy may change as the bubble population becomes denser. Power- and frequency-dependent studies have reported non-proportional or non-monotonic changes in sonochemical output, while reactor height, geometry, and acoustic-field organization can decouple chemical response from calorimetric dissipation [[13], [14], [15], [16], [17], [18], [19]]. Interbubble coupling, shielding, scattering, coalescence, pressure-field redistribution, and increased partitioning into heat or streaming can therefore modify the incremental chemical return [[3], [14], [20]]. The important question is not simply whether a dosimetric signal increases with power, but whether chemically distinct reporters scale in the same way under the same calorimetrically defined perturbation.

The literature thus establishes that absolute sonochemical yields depend on both the reporter and the operating state, but it does not resolve whether disagreement among reporters is a constant multiplicative offset or changes along a matched power trajectory. If the difference were only a fixed assay-specific factor, within-probe normalized power-response curves would coincide. Divergence of those curves instead means that changing power redistributes the measurable chemical response among reaction channels. We did not identify a previous study that tested this distinction by applying a common calorimetric power series to KI/I_3_^−^, H_2_O_2_, Fricke/Fe^3^⁺, and aromatic hydroxylation in the same reactor. This matched comparison, rather than a new dosimetry reaction, defines the specific gap addressed here.

Dissolved gas provides an orthogonal perturbation because it changes both bubble physics and bubble chemistry. Gas solubility and diffusivity influence nuclei availability and gas exchange, while the heat-capacity ratio, thermal conductivity, and molecular degrees of freedom affect collapse thermodynamics. Gas molecules can also participate in high-temperature intrabubble reactions [[21], [22], [23], [24], [25]]. Argon is effectively inert and commonly favors strong compression heating, whereas N_2_ can reduce the collapse temperature relative to Ar and can enter nitrogen-oxide chemistry. Gas-dependent H_2_O_2_ accumulation is therefore useful for testing whether a measured response is governed by energy input alone or by the chemical identity of the bubble environment.

The Meinhardt multifrequency platform used here has previously been applied to frequency-dependent sonochemistry and to a dedicated study of peroxynitrite formation [26]. To maintain a clear publication boundary, the previously published frequency- and ONOO⁻-dependent datasets are not re-plotted or treated as primary results in the present manuscript. The analysis instead focuses on the complete 585 kHz power series and uses the gas experiment only as a complementary perturbation of the H_2_O_2_ channel.

Accordingly, this work develops power-response fingerprinting (PRF), in which each dosimetry method is treated as one coordinate of a chemical response vector rather than as an interchangeable estimate of a single radical pool. KI/I_3_⁻, H_2_O_2_, Fricke/Fe^3^⁺, and 4-NP/4-NC are compared through formation rates, energy-specific responses, within-probe normalization, and descriptive power-response metrics. The contribution is not a new dosimetry reaction, an independently measured bubble population, or a universal power law. It is a matched, probe-aware test of whether the relative expression of four measurable chemical channels is preserved when calorimetrically measured power changes. The objective is therefore to determine whether probe divergence contains quantitative information about reactor-state changes that would be hidden by a single dosimetry method or an absolute-yield comparison.

## 2 Materials and methods

### 2.1 Chemicals and high-frequency sonoreactor

Potassium iodide (KI, 99%), sodium chloride (NaCl, 99.5%), sodium hydroxide (NaOH, 99%), and 4-nitrophenol (4-NP, 99.5%) were obtained from Sigma-Aldrich. Ferrous ammonium sulfate hexahydrate (Mohr salt, Fe(NH_4_)_2_(SO_4_)_2_·6H_2_O, 99%) was supplied by Panreac, sulfuric acid (96–98%) by Prolabo, and ammonium heptamolybdate tetrahydrate (AHM, 99%) by Sigma-Aldrich. All solutions were prepared with ultrapure water and reagents were used without further purification.

Experiments were carried out in a 500 mL jacketed glass reactor coupled at its base to a Meinhardt Ultraschalltechnik multifrequency transducer with an active diameter of 5.3 cm. The platform can operate at 585, 860, and 1140 kHz and has been described previously [26]. Based on the previously reported multifrequency comparison for this reactor [26], the present PRF analysis is intentionally restricted to 585 kHz, which exhibited the highest energy-specific sonochemical response among the investigated frequencies. This restriction isolates the effect of acoustic power while avoiding re-presentation of the previously published frequency-dependent dataset.

Unless otherwise stated, the working volume was 300 mL and the liquid temperature was maintained at 25 ± 2 °C by recirculating thermostated water through the reactor jacket. Solutions used for the three-power PRF matrix were not deliberately sparged and were handled under ambient laboratory atmosphere. Concentration-time profiles were followed for 60 min; slopes were evaluated over the linear response interval of each assay.

### 2.2 Calorimetric acoustic power

The calorimetric acoustic power dissipated in the liquid was determined from the initial linear temperature rise during sonication. Over the short calorimetric interval, heat loss to the surroundings was minimized and the integral power balance was calculated as:

(1) $$P_{cal}=mC_{p}{\left(\frac{dT}{dt}\right)}_{0}$$

where *P_cal_* is the calorimetric acoustic power (W), *m* is the liquid mass (g), *C_p_* is the specific heat capacity of water (J g⁻^1^ K⁻^1^), and (d*T*/d*t*)_0_ is the initial temperature-rise rate (K s⁻^1^). At 585 kHz, amplitude settings 2, 3, and 4 corresponded to *P_cal_* = 9.65, 38.0, and 79.0 W, respectively (Table 1). The active transducer area, calculated from its 5.3 cm diameter, was 22.06 cm^2^. Division of *P_cal_* by this area yielded area-normalized calorimetric powers, *P_cal_*/*A*, of 0.44, 1.72, and 3.58 W cm⁻^2^. These quantities are area-normalized power descriptors, not spatially resolved acoustic intensities. The dimensionless power ratio *Π*(*P*) = *P_cal_*/*P_cal,low_*, with *P_cal,low_* = 9.65 W, was 1.00, 3.94, and 8.19. All PRF calculations use *P_cal_* rather than nominal generator amplitude. Calorimetry supplies an integral energy denominator but does not partition direct acoustic absorption, viscous and streaming losses, bubble-related dissipation, or heat conducted from the transducer-reactor assembly; it is therefore not identified with chemically productive cavitation power.

**Table 1:** Calorimetric power levels used for the 585 kHz PRF matrix.

**Amplitude setting**	***P_cal_* (W)**	***P_cal_*/*A* (W cm⁻^2^)**	***Π*(*P*) = *P_cal_*/*P_cal,low_***
2	9.65	0.44	1.00
3	38.0	1.72	3.94
4	79.0	3.58	8.19

*P_cal,low_* = 9.65 W. The active transducer area was 22.06 cm^2^, calculated from its 5.3 cm diameter; *P_cal_*/*A* is reported as an area-normalized calorimetric power and not as a spatially resolved acoustic intensity.

### 2.3 Potassium iodide dosimetry

KI solutions (0.10 M; 300 mL) were irradiated for 60 min at each selected acoustic-power condition without deliberate gas sparging. Cavitation-derived oxidants convert iodide to iodine-containing intermediates that yield triiodide in the presence of excess iodide. I_3_⁻ was quantified spectrophotometrically at 353 nm using a 1 cm quartz cuvette and ε = 26,300 L mol⁻^1^ cm⁻^1^. Because the Weissler-type iodide response integrates several oxidation routes, the measured I_3_^−^ formation rate is treated as a dosimetric response rather than as a stoichiometrically unique measure of HO^•^ production [4], [5], [6], [7].

### 2.4 Hydrogen peroxide determination

H_2_O_2_ produced during sonication of water was determined iodometrically in experiments separate from the 0.10 M KI dosimetry described in Section 2.3. A 200 µL aliquot of sonicated water was mixed after withdrawal with 1.0 mL of KI solution (0.10 M) and 20 µL of ammonium heptamolybdate solution (0.010 M). After 5 min, the I_3_⁻ absorbance generated by H_2_O_2_ oxidation of iodide was measured at 353 nm (ε = 26,300 L mol⁻^1^ cm⁻^1^). The directly sonicated KI response from Section 2.3 was therefore not present in this aliquot and was not subtracted. Transient HO^•^ does not persist through withdrawal and the 5 min post-sonication assay; the analytical response is assigned to accumulated H_2_O_2_ under the iodometric conditions. H_2_O_2_ is interpreted as a stable molecular outcome of the radical network, while its measured concentration represents net accumulation after simultaneous formation and consumption processes [[7], [14]].

### 2.5 Fricke dosimetry

The Fricke solution contained Fe(NH_4_)_2_(SO_4_)_2_·6H_2_O (1.0 mM), H_2_SO_4_ (0.40 M), and NaCl (1.0 mM). Sonochemically generated Fe3⁺ was quantified at 303 nm using ε = 2197 L mol⁻1 cm⁻1. Because Fe2⁺ oxidation proceeds through coupled radical and molecular pathways, including contributions from HO^•^, HO_2_^•^/O_2_^•^⁻ chemistry, and H_2_O_2_, the Fe3⁺ signal is treated as an integrated aqueous oxidation response rather than as a species-specific HO^•^ assay [5], [6], [7].

### 2.6 4-Nitrophenol hydroxylation dosimetry

A 4-NP solution (0.139 g L⁻1, approximately 1.0 mM) was adjusted to pH 5 and irradiated in a 300 mL volume. Samples of 1.5 mL were periodically withdrawn and mixed with an equal volume of 0.20 M NaOH before UV–visible analysis. The parent 4-NP absorbs near 399 nm, whereas the hydroxylation product 4-nitrocatechol (4-NC) was quantified at 510 nm using ε = 12,400 L mol⁻1 cm⁻1. The 4-NC formation rate is used as a marker of an aromatic hydroxylation channel and is not equated to the total HO^•^ yield because radical addition can generate multiple products and 4-NC itself can undergo further oxidation [[6], [8]].

### 2.7 Gas-conditioning experiments

Gas effects on H_2_O_2_ accumulation were evaluated at 585 kHz using amplitude setting 4, corresponding to *P_cal,ref_* = 79 W under the reference calorimetric condition. H_2_O_2_ was selected for this focused gas perturbation because it can be followed as a stable net product in water without adding a concentrated dosimetry matrix during sonication; extending the comparison across chemically different probe solutions would conflate gas effects with matrix-specific bubble and reaction chemistry. A 300 mL water volume was either used without deliberate gas sparging (unsparged water) or conditioned with air, Ar, or N_2_, and H_2_O_2_ accumulation was followed for 60 min using the iodometric method described above. Gas-specific calorimetry was not independently determined for each state. The gas comparison is therefore an operational response at a common generator setting, not a comparison at rigorously identical gas-resolved calorimetric power.

The gas-conditioning experiments constituted a separate experimental series from the three-power PRF matrix. The air- and Ar-conditioned H_2_O_2_ values were reported previously in Ref. [26]; the unsparged and N_2_ states complete the present four-condition comparison. Gas effects were evaluated only by within-series normalization to the unsparged-water reference through *M_g_*, and gas-series rates were not pooled with or treated as replicates of the H_2_O_2_ rates in the PRF power series.

### 2.8 Power-response fingerprinting and quantitative analysis

For each dosimetry method *i*, the apparent formation rate *r_i_* was obtained by least-squares regression of concentration against sonication time over the stated 60 min response window, restricted to the linear interval when required:

(2) $$r_{i}=\frac{{dC}_{i}}{dt}$$

To distinguish absolute chemical productivity from the response generated per unit calorimetric energy, the energy-specific dosimetric response was calculated as:

(3) $$G_{i}=\frac{r_{i}V}{P_{cal}}$$

where *V* is the irradiated volume. Before applying Eq. (3), *r_i_* was converted from µmol L⁻^1^ min⁻^1^ to mol L⁻^1^ s⁻^1^, so that *G_i_* is obtained in mol J⁻^1^ and is reported as nmol J⁻^1^ for readability. *G_i_* is deliberately termed an energy-specific dosimetric response rather than an absolute radical G-value. Because the assays differ in reaction stoichiometry, solution composition, and selectivity, numerical *G_i_* values are interpreted within each probe and are not used to rank primary-radical production across different dosimetries.

The sensitivity of each measured channel to acoustic power was summarized using the empirical relation:

(4) $$r_{i}=A_{i}P_{cal}^{\beta_{i}}$$

where *A_i_* is an empirical coefficient and *β_i_* is the apparent global power-response exponent determined by log–log regression. *β_i_* = 1 denotes proportional scaling with calorimetric power, *β_i_* < 1 sub-proportional scaling, and *β_i_* > 1 super-proportional scaling. Because only three power levels were investigated, *β_i_* is used strictly as a descriptive PRF coordinate over the 9.65–79 W window and not as a kinetic order or universal scaling law.

Two within-probe dimensionless descriptors were then defined. The output ratio, which measures the gain in chemical productivity relative to the low-power state, is:

(5) $$R_{i}\left(P_{cal}\right)=\frac{r_{i}\left(P_{cal}\right)}{r_{i}\left(P_{cal,low}\right)}$$

and the energy-efficiency retention, which measures the fraction of the low-power response per joule that remains at power *P*, is:

(6) $$\Phi_{i}\left(P_{cal}\right)=\frac{G_{i}\left(P_{cal}\right)}{G_{i}\left(P_{cal,low}\right)}$$

with *P_cal,low_* = 9.65 W. Because the irradiated volume is fixed, Eqs. (3), (5), and (6) give the identity *Φ_i_*(*P*) = *R_i_*(*P*)/*Π*(*P*), where *Π*(*P*) = *P_cal_*/*P_cal,low_*. *R_i_* and *Φ_i_* are algebraically related views of the same rate-power data. They are displayed together in Fig. 1 to juxtapose output gain and retained response per joule; no independent information is claimed. Gas-conditioned H_2_O_2_ responses were expressed using the gas-modulation factor, *M_g_*:

(7) $$M_{g}=\frac{r_{g}}{r_{unsparged}}$$

where *r_g_* is the H_2_O_2_ formation rate under gas *g* and *r_unsparged_* is the corresponding rate in unsparged water. Linear least-squares regression was used for concentration–time slopes and for the three-point log–log estimate of *β_i_*. Coefficients of determination (*R*^2^) are reported as descriptive measures of fit quality.

**Fig. 1:**
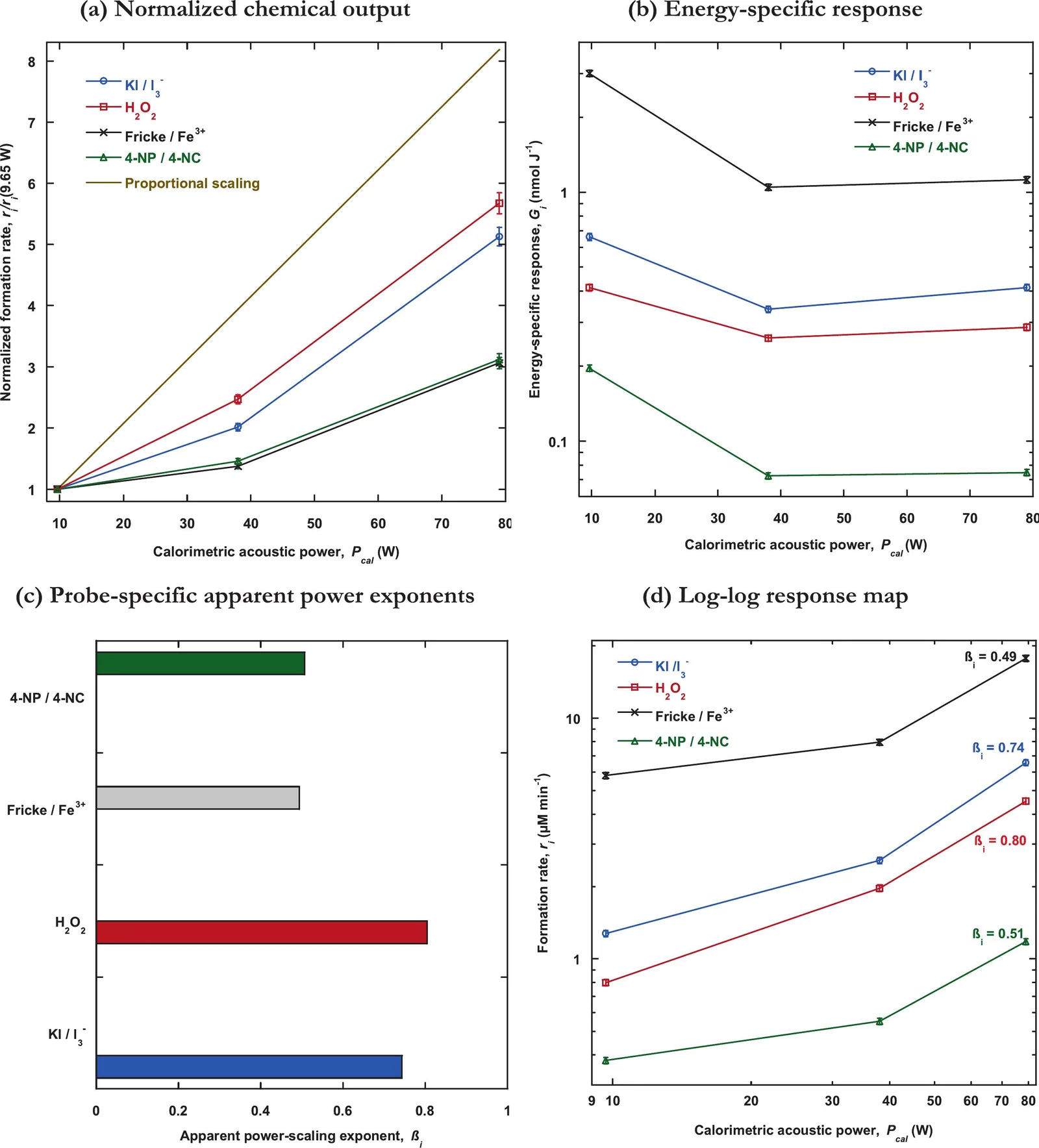
Power-response fingerprinting at 585 kHz for unsparged solutions. (a) Formation rates normalized to the 9.65 W value; the dashed line denotes proportional scaling, *Π*(*P*) = *P_cal_*/*P_cal,low_*. (b) Energy-specific dosimetric response *G_i_* versus *P_cal_*. (c) Descriptive global power-response exponents *β_i_* obtained from *r_i_* = *A_i_P_cal_*^βi^; the dashed line marks *β* = 1. (d) Log-log rate-power map. The *β_i_* values characterize only the 9.65–79 W window and are not kinetic orders or universal scaling laws. Error bars in panels (a), (b), and (d) show conservative ± 3% limits; the observed replicate experimental error was below 3%. No inferential comparison is claimed.

All derived quantities (*G_i_*, *R_i_*, *Φ_i_*, *β_i_*, and *M_g_*) were calculated from the unrounded regression slopes and calorimetric power values; numerical values were rounded only for presentation.

Replicate measurements were obtained for each operating condition, and the relative experimental error was below 3% for all reported responses. Conservative ± 3% error limits are displayed for the experimental points in Fig. 1(a,b,d) and 3; the plotted limits therefore encompass the observed replicate variability. The reported *R*^2^ values quantify the linearity of the concentration–time regressions and are not measures of replicate precision. No formal hypothesis testing was performed, and cross-condition comparisons are interpreted descriptively.

## 3 Results and discussion

### 3.1 A common calorimetric power axis exposes non-equivalent chemical rates

A rate-based comparison first requires that product concentration vary sufficiently linearly over the analyzed time interval. For the unsparged three-power matrix, the concentration–time regressions gave *R*^2^ = 0.993–0.997 for I_3_⁻, 0.938–0.987 for H_2_O_2_, 0.990–1.000 for Fe^3^⁺, and 0.995–0.999 for 4-NC (Table 2). These ranges summarize the regressions at the three power levels for each probe. Representing each assay by its slope avoids arbitrary endpoint selection and places chemically different reporters on the same experimentally measured *P_cal_* axis. Replicate experimental error was below 3% for every reported response; *R*^2^ assesses kinetic linearity rather than replicate precision.

**Table 2:** Global power-response fingerprint parameters at 585 kHz for unsparged solutions handled under ambient laboratory atmosphere.

**Probe**	***r*_9.65_**	***r*_38_**	***r*_79_**	***β***	***R*^2^*_β_***	***G*_9.65_**	***G*_38_**	***G*_79_**
KI/I_3_⁻	1.277	2.571	6.547	0.74	0.936	0.662	0.338	0.414
H_2_O_2_	0.798	1.971	4.531	0.80	0.978	0.414	0.259	0.287
Fricke / Fe^3^⁺	5.790	7.955	17.733	0.49	0.835	3.000	1.047	1.122
4-NP / 4-NC	0.379	0.551	1.181	0.51	0.871	0.196	0.073	0.075

Formation rates *r* are in µM min⁻^1^; *G* values are within-assay energy-specific responses in nmol J⁻^1^. The concentration–time *R*^2^ ranges at 9.65, 38.0, and 79.0 W were 0.993–0.997 (KI/I_3_^−^), 0.938–0.987 (H_2_O_2_), 0.990–1.000 (Fricke/Fe^3^⁺), and 0.995–0.999 (4-NP/4-NC). *β* and *R*2*_β_* refer to the three-point log–log regressions and are descriptive PRF coordinates, not kinetic orders. Replicate experimental error was below 3% for all reported rates.

Formation rates increased with *P_cal_* for every probe, but the increase was not proportional and differed among probes (Table 2). An 8.19-fold rise in calorimetric power produced gains of 5.13-fold for KI, 5.67-fold for H_2_O_2_, 3.06-fold for Fricke, and 3.12-fold for 4-NC. Thus, the matched experiment identifies a change in the relative expression of the four measured channels, not merely a uniform increase in chemistry.

### 3.2 Power-response exponents reveal probe-specific sub-proportional scaling

Fig. 1(a) compares the observed normalized rates with the proportional response expected from *Π*(*P*) = *P_cal_*/*P_cal,low_*. None of the four assays reaches the 8.19-fold rate increase expected for proportional scaling at 79 W. The descriptive global exponents separate H_2_O_2_ and KI (*β_i_* = 0.80 and 0.74) from 4-NC and Fricke (*β_i_* = 0.51 and 0.49) (Fig. 1(c,d)). Within the investigated window, H_2_O_2_ accumulation and iodide oxidation retain a larger fraction of the incremental power response than the Fricke and aromatic-hydroxylation channels. This ranking concerns response scaling and does not rank absolute radical yields.

Sub-proportional global scaling is physically plausible in a multibubble field. Increasing acoustic power can activate additional nuclei and increase bubble excursion, but a denser population also strengthens interbubble coupling, shielding, scattering, and attenuation and can redirect energy toward streaming and heat [[3], [14], [20]]. Bubble-bubble interactions can suppress cavitation-driven physicochemical effects [20], KI reaction rate can decouple from calorimetric dissipation across reactor geometries [15], and power-induced changes in wave superposition and bubble organization can generate distinct reaction regimes [16]. *P_cal_* is therefore an appropriate integral energy denominator, but it is not a direct measurement of chemically productive cavitation power.

Two high-power mechanisms identified in multibubble studies are especially relevant. First, Hatanaka et al. observed that multibubble sonoluminescence increased with power and then quenched when mobile millimetre-scale bubble clusters appeared; frequent coalescence driven by secondary Bjerknes attraction can create cluster populations that are weakly sonochemically active [[27], [28], [29]]. Second, at excessive acoustic amplitude, the primary Bjerknes force can repel strongly oscillating bubbles from pressure antinodes, shortening their residence in regions of high acoustic amplitude and reducing the population of effective collapses [[27], [29]]. Either mechanism can lower chemical output per joule while total calorimetric dissipation continues to rise. The present rates continue to increase, so the data demonstrate sub-proportional scaling rather than complete quenching. Because bubble imaging and pressure-field mapping were not performed, cluster formation and antinode repulsion remain mechanistic hypotheses rather than directly observed causes in this 585 kHz reactor.

H_2_O_2_ is a net molecular accumulation product arising substantially from recombination of water-derived radical intermediates. Increasing instantaneous radical availability can favor bimolecular recombination, but simultaneous H_2_O_2_ formation and consumption mean that the measured slope remains a net pathway response rather than a direct measure of primary-radical production. The concentrated iodide matrix efficiently intercepts a broad network of oxidizing intermediates. Fricke dosimetry, by contrast, is a chain oxidation system in a strongly acidic Fe^2^⁺ matrix, while 4-NC is only one measurable product branch within the broader oxidation network of 4-NP [[5], [6], [7], [8]]. The *β_i_* values therefore combine cavitation-field evolution with assay-specific reaction and transport chemistry; they should not be interpreted as intrinsic exponents for primary HO^•^ generation.

### 3.3 Energy normalization reveals a productivity-efficiency trade-off

Absolute formation rates were highest at 79 W, whereas the energy-specific response fell sharply from 9.65 to 38 W and recovered only modestly at 79 W (Fig. 1(b), Table 2). At the high-power state, the retained response per joule was 0.63 for KI, 0.69 for H_2_O_2_, 0.37 for Fricke, and 0.38 for 4-NC relative to the corresponding 9.65 W values. Maximum chemical flux and maximum response per unit dissipated energy therefore occurred at different operating states. More generally, prior single-bubble measurements have shown that acoustic energy is partitioned among photons, radicals, and ions with distinct efficiencies [30].

The engineering implication is that productivity and energy-specific performance favor different tested operating states. Among the three power levels examined, 79 W gave the highest measured product flux for every assay, whereas 9.65 W gave the highest measured response per joule of dissipated energy. Within-probe normalization is essential: absolute *G_i_* values cannot be ranked across assays because the Fricke chain reaction, KI oxidation network, H_2_O_2_ accumulation, and 4-NC product branch have different stoichiometries and matrices. The separation of H_2_O_2_/KI from Fricke/4-NC at 79 W identifies channels with similar power sensitivity; it does not establish identical elementary species or reaction zones.

### 3.4 Paired dimensionless maps compare output gain and efficiency retention

Fig. 2 presents the same rate-power matrix in two dimensionless views. *R_i_*(*P*) shows the gain in measured chemical output relative to 9.65 W, whereas *Φ_i_*(*P*) shows the fraction of the low-power response per joule that remains. The panels are algebraically related through *Φ_i_*(*P*) = *R_i_*(*P*)/*Π*(*P*); they are retained as a compact visual comparison, not as independent evidence. At 79 W, *R_i_* = [5.13, 5.67, 3.06, 3.12] and *Φ_i_* = [0.63, 0.69, 0.37, 0.38] for KI, H_2_O_2_, Fricke, and 4-NC, respectively.

**Fig. 2:**
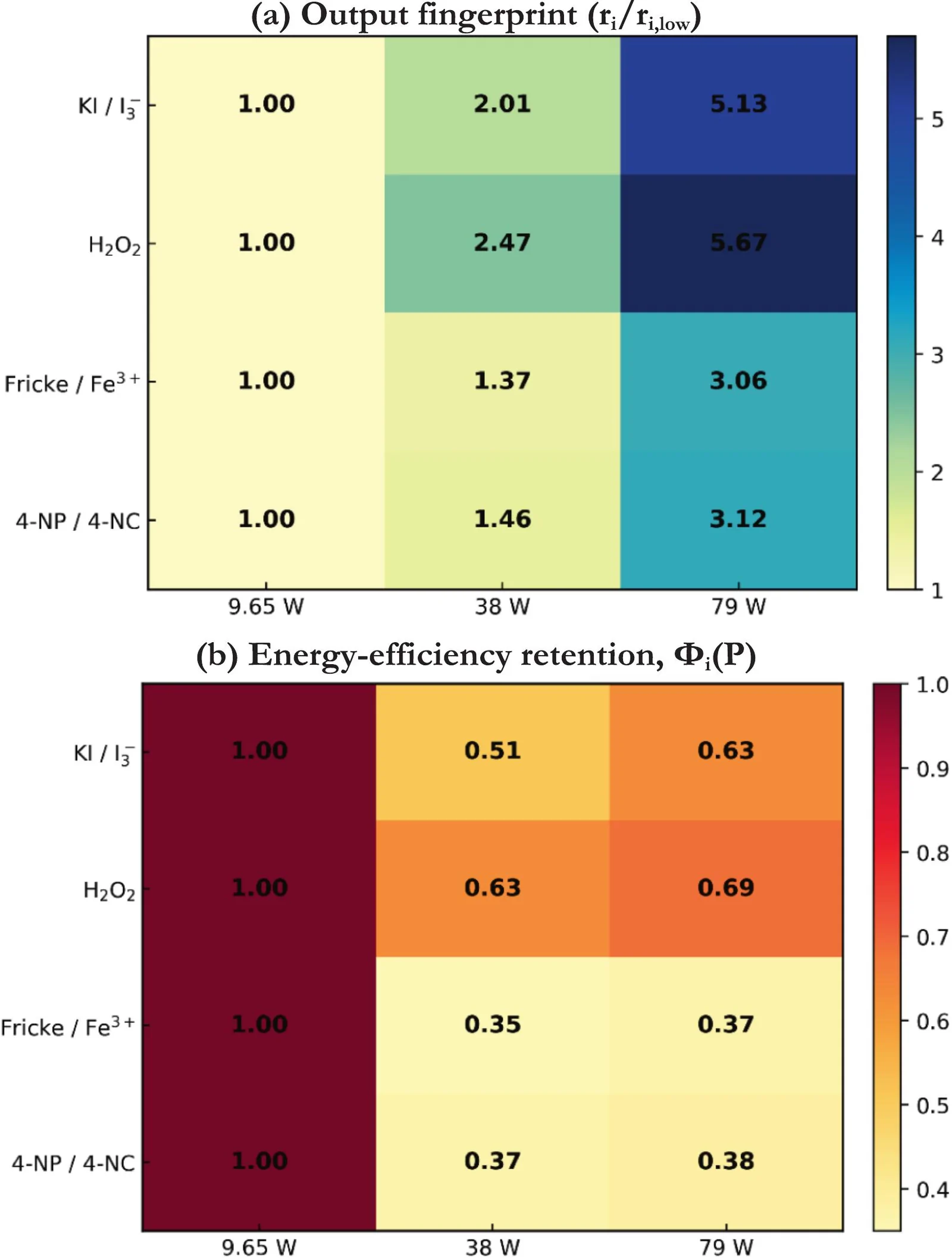
Paired dimensionless maps at 585 kHz. (a) Within-probe output gain *R_i_*(*P*) = *r_i_*(*P*)/*r_i,low_*. (b) Energy-efficiency retention *Φ_i_*(*P*) = *G_i_*(*P*)/*G_i,low_*. Both panels are normalized to 9.65 W and are algebraically related through *Φ_i_* = *R_i_*/*Π*; they are shown together to compare chemical productivity with retained response per joule. Values were calculated from the mean replicate responses, for which the experimental error was below 3%, and are interpreted descriptively.

Normalization is applied within each probe. Absolute *G_i_* values cannot be compared across assays because their matrices, selectivities, and stoichiometries differ.

The paired map shows that H_2_O_2_ and KI retain about two-thirds of their low-power response per joule at 79 W, whereas Fricke and 4-NC retain only 0.37–0.38. This grouping describes response scaling only and does not prove common elementary species or reaction zones.

### 3.5 Gas composition selectively redirects the H_2_O_2_ accumulation channel

Gas conditioning provides a chemically distinct perturbation of the H_2_O_2_ channel. At the common high-power generator setting, Ar gave the largest H_2_O_2_ formation rate and N_2_ the smallest (Fig. 3). Relative to unsparged water, the Ar response was 1.88-fold, whereas the N_2_ response was 0.16-fold; the Ar/N_2_ rate ratio was 11.4. These values demonstrate a strong gas effect without implying equal gas-resolved calorimetric power. The 3.93 µM min⁻^1^ unsparged rate belongs to this dedicated gas-conditioning campaign, whereas the 4.531 µM min⁻^1^ value in Table 2 belongs to the separate PRF power campaign. They are not replicate measurements and were neither pooled nor compared statistically; *M_g_* was calculated only within the gas series.

**Fig. 3:**
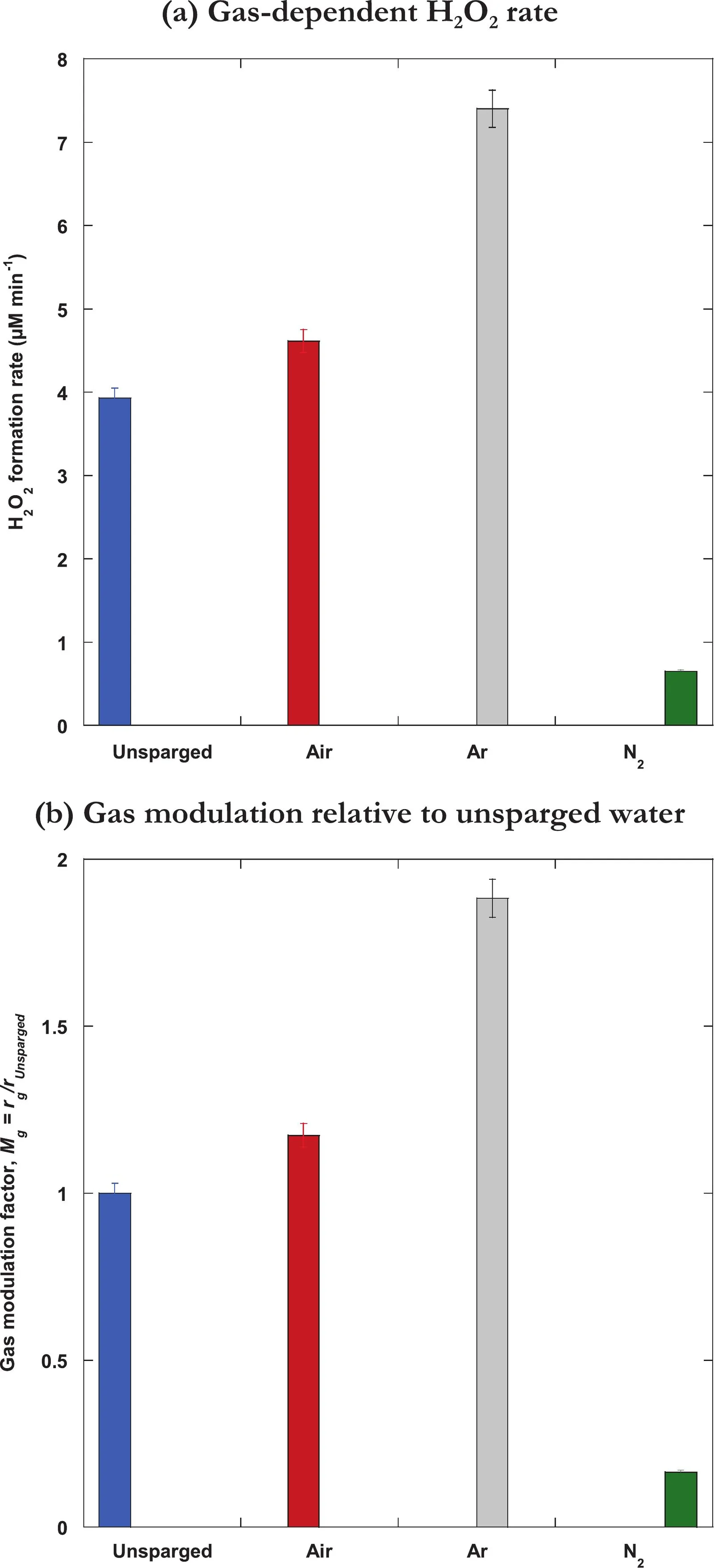
Gas-dependent H_2_O_2_ response at 585 kHz and amplitude setting 4 (reference P_cal,ref_ = 79 W). (a) Apparent formation rates from linear concentration–time profiles and (b) gas-modulation factor M_g_ relative to unsparged water. Air and Ar (asterisks and hatched bars) are previously published data from Ref. [26]; the unsparged and N_2_ states complete the present gas comparison. Gas-specific calorimetric powers were not measured. Error bars show conservative ± 3% limits; the observed replicate experimental error was below 3%.

The Ar enhancement and N_2_ suppression are consistent with coupled thermophysical and chemical effects. Relative to monatomic Ar, diatomic N_2_ has a lower heat-capacity ratio and additional rotational and vibrational energy-storage modes, which increase thermal damping and can lower the collapse temperature. Numerical analysis predicts an optimum bubble temperature of approximately 5500 K for oxidant production: lower temperatures limit water–vapor dissociation, whereas higher temperatures intensify radical-consuming reactions [24]. The gas-dependent rate cannot therefore be interpreted from heat-capacity ratio alone, but the lower collapse severity expected for N_2_ relative to Ar provides one plausible contribution.

Chemical diversion provides a second contribution. For sufficiently hot N_2_-containing bubbles, OH and O-derived oxidants can be consumed in reactions that oxidize nitrogen, with NO, HNO_2_, and HNO_3_ among the predicted products [25]. This pathway competes with escape and recombination of water-derived radicals to H_2_O_2_. The absence of O_2_ is not, by itself, an adequate explanation because Ar sparging would also be expected to substantially displace dissolved O_2_ yet produced the highest H_2_O_2_ rate. O_2_ can influence secondary HO_2_^•^/O_2_^•⁻^ chemistry, but the observed Ar-N_2_ contrast points to the combined effects of collapse thermodynamics, nitrogen chemistry, gas transfer, bubble population, and gas-dependent energy coupling. The present measurements do not resolve their individual contributions.

The modest difference between air-conditioned and unsparged water is consistent with as-prepared laboratory water already containing atmospheric gases, so air sparging is a smaller compositional perturbation than replacement with pure Ar or N_2_. *M_g_* is consequently an operational gas-response factor at the selected generator setting, not a chemical selectivity measured at identical gas-specific *P_cal_*. This distinction is necessary because gas identity can alter both bubble chemistry and energy coupling to the liquid [[21], [31]].

The air- and Ar-conditioned values in Fig. 3 were reported previously in Ref. [26] and are marked explicitly as previously published data in the revised caption. They are included only to complete the four-condition gas comparison at the same nominal generator setting. The unsparged and N_2_ values are from the present analysis. No previously published frequency-dependent or ONOO⁻ figure is reproduced as a new result.

### 3.6 PRF reframes cavitation as a chemical response vector

Taken together, the power and gas perturbations support a response-vector description of high-frequency sonochemistry. An operating sonoreactor does not possess one chemically universal “intensity” that every assay samples proportionally. Rather, each state is represented by a vector whose coordinates depend jointly on the cavitation field and the reaction matrix. Increasing *P_cal_* moves all four absolute-rate coordinates upward, but by different factors; energy normalization moves all four below the low-power baseline, again by different amounts. Changing the bubble gas then redistributes the H_2_O_2_ coordinate over an order-of-magnitude range. The chemically relevant object is therefore a multidimensional response surface indexed by operating condition and reporter, not a single radical-yield number.

This interpretation is consistent with prior observations from the same reactor family and with independent multibubble studies. Earlier multifrequency measurements showed that the energy-specific responses of conventional probes decreased with increasing frequency, although the magnitude was probe dependent [26]. Interbubble interaction can suppress cavitation-driven physicochemical effects [20], KI activity can decouple from calorimetric dissipation across reactor geometries [15], and power-induced reorganization can generate distinct reaction regimes [16]. At excessive power, bubble-cluster formation and primary-Bjerknes repulsion from pressure antinodes provide two established routes to sonochemical quenching [[27], [28], [29]]. PRF gives these physical mechanisms a matched chemical constraint without claiming to resolve bubble dynamics directly.

Fig. 4 summarizes the mechanistic interpretation supported by the present data. Increasing power is expected to move the reactor from a relatively sparse active field toward a denser, more strongly interacting multibubble population. Cluster formation can create weakly active bubble populations, and primary Bjerknes forces can displace bubbles from pressure antinodes at excessive amplitude [[27], [28], [29]]. These mechanisms, together with shielding and non-chemical dissipation, can reduce the incremental chemical return. The global *β_i_* values are macroscopic descriptors of the resulting trajectory. They cannot identify bubble number, collapse temperature, or spatial reaction zones uniquely, but they define quantitative constraints that future acoustic-field measurements and bubble-dynamics models should reproduce.

**Fig. 4:**
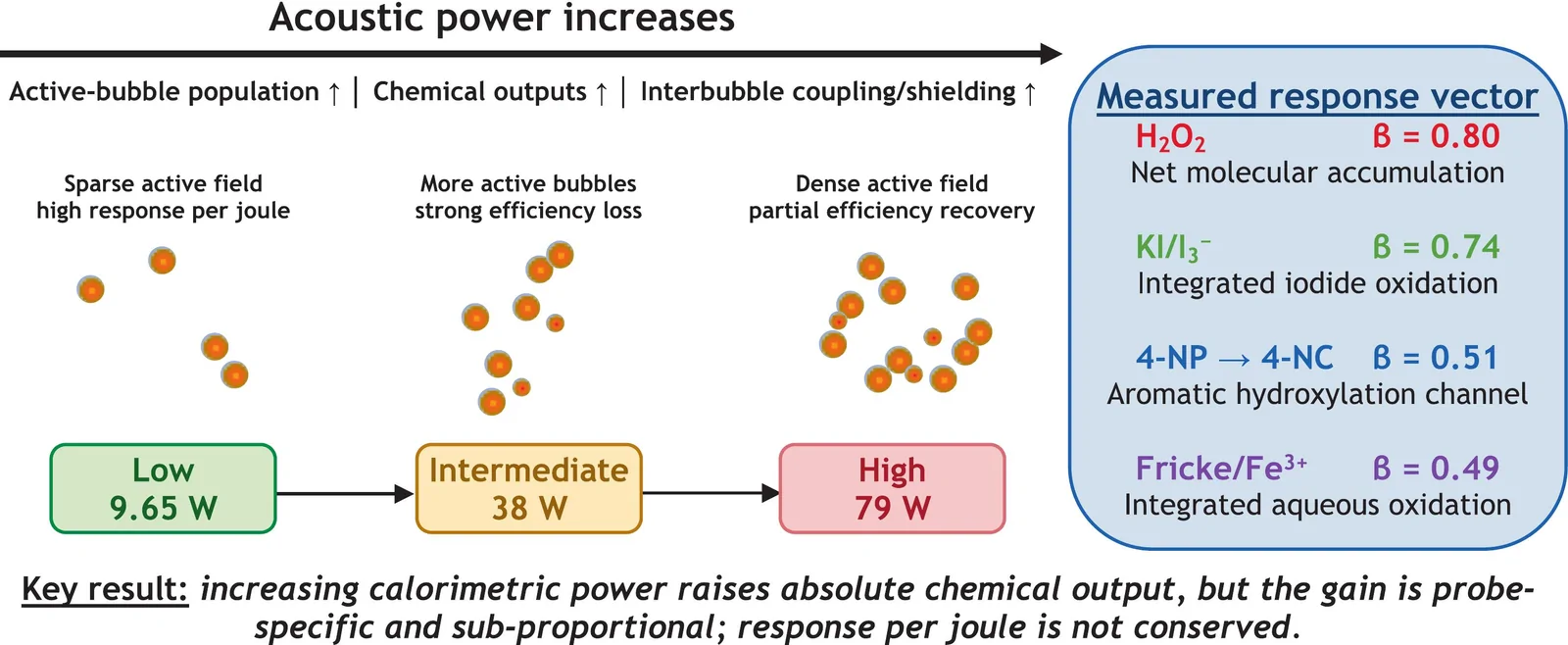
Mechanistic synthesis of the PRF interpretation. Increasing calorimetric power can increase active-bubble population and absolute chemical production while strengthening interbubble coupling, shielding, bubble-cluster formation, primary-Bjerknes displacement from pressure antinodes, and non-chemical dissipation [[27], [28], [29]]. Global β_i_ values summarize full-window response scaling. The conceptual bubble populations and pathways are not direct measurements.

### 3.7 Implications for reactor characterization and scale-up

The results suggest a practical hierarchy for reporting and comparing sonochemical reactors. First, nominal amplitude should be accompanied by calorimetric power because electrical settings are equipment specific [4]. Second, *P_cal_* is an integral measure of energy converted to heat in the monitored system; it cannot distinguish acoustic absorption, viscous and streaming losses, bubble-related dissipation, or thermal leakage from the transducer-reactor assembly. A rise in *P_cal_* can therefore occur without a proportional rise in chemically effective cavitation [[15], [31]]. Third, a single chemical dosimetry method should not be generalized beyond its reaction matrix. Multiple chemically distinct reporters should be compared on the same measured power basis and normalized within probe when mechanistic interpretation is required.

PRF operationalizes this hierarchy without forcing incompatible assays onto a common radical scale. The absolute rate vector describes chemical productivity, *Φ_i_* quantifies the retained response per joule, and *β_i_* summarizes global power sensitivity over a defined window. These descriptors complement rather than replace hydrophone measurements, acoustic-pressure mapping, sonochemiluminescence imaging, or validated bubble-dynamics calculations when a spatially resolved mechanism is required.

For scale-up, the sub-unity global *β_i_* values caution against assuming linear conversion of installed acoustic power into chemical throughput. Extrapolation beyond the present 9.65–79 W range would be unjustified because the energy-specific responses change nonlinearly over the measured range. A change in PRF with scale, geometry, gas environment, or frequency could nevertheless serve as a sensitive indicator of altered energy partitioning or bubble-population dynamics. Coupling PRF with acoustic diagnostics in future work would therefore provide a direct route from reactor-scale chemical performance to the underlying cavitation physics.

## 4 Conclusions

Power-response fingerprinting shows that chemically distinct sonochemical dosimetries do not behave as scaled replicas when calorimetric acoustic power is increased. At 585 kHz, raising *P_cal_* from 9.65 to 79 W increased the KI, H_2_O_2_, Fricke, and 4-NC formation rates by 5.13-, 5.67-, 3.06-, and 3.12-fold, respectively, despite an 8.19-fold increase in dissipated power. The corresponding descriptive global exponents were 0.74, 0.80, 0.49, and 0.51. The matched normalized responses therefore diverge with power; the differences are not fixed assay-specific offsets.

Energy normalization shows that the response per joule decreased most strongly between 9.65 and 38 W and recovered only partly at 79 W. At 79 W, all four responses remained below their low-power baselines. Maximum absolute productivity and maximum energy-specific response therefore occurred at different operating states.

Gas conditioning independently demonstrates the sensitivity of the H_2_O_2_ response to the gas-conditioned reactor state: Ar increased H_2_O_2_ accumulation to 1.88 times the unsparged-water rate, whereas N_2_ reduced it to 0.16 times that rate. The contrast cannot be attributed solely to dissolved-O_2_ displacement because Ar sparging would also be expected to substantially displace O_2_. The result is consistent with combined differences in collapse thermodynamics, nitrogen-oxidation chemistry, gas transfer, bubble-population dynamics, and gas-dependent energy coupling.

Uncited reference

[30].

## CRediT authorship contribution statement

**Oualid Hamdaoui:** Investigation; Conceptualization; Data curation; Methodology; Formal analysis; Resources; Validation; Project administration; Supervision; Funding acquisition; Visualization; Writing original draft; Writing - review & editing. **Hamza Ferkous:** Investigation; Data curation; Validation; Visualization. **Muthupandian Ashokkumar:** Conceptualization; Validation; Supervision; Visualization.

## Declaration of competing interest

The authors declare that they have no known competing financial interests or personal relationships that could have appeared to influence the work reported in this paper.
